# Supplementary material for: Prognostic nutritional index in risk of mortality following fulminant myocarditis
Source: Sci Rep. 2025 Nov 21;15:41379. doi: 10.1038/s41598-025-25385-7 (PMC12638857; doi:10.1038/s41598-025-25385-7)
Supplement: Supplementary file 1 — Supplementary Material 1 [file 41598_2025_25385_MOESM1_ESM.docx]

**Supplementary Table 1. Characteristics of fulminant myocarditis on admission.**

|  | **Patients with PNI>40 on admission (n=177)** | | **Patients with PNI≤40 on admission (n=146)** | |  |
| --- | --- | --- | --- | --- | --- |
| **Characteristics** | **Patients with available data** | **Values** | **Patients with available data** | **Values** | ***p* value** |
| Demographic findings | | | | | |
| Age, y | 177 | 46 (33–62) | 146 | 54 (40–67) | 0.0020 |
| Female | 177 | 68 (38) | 146 | 75 (44) | 0.0196 |
| BMI on admission, kg/m^2^ | 173 | 22.5 (20.1–25.0) | 142 | 21.5 (19.3–23.6) | 0.0242 |
| NYHA Ⅲ or Ⅳon admission | 169 | 135 (80) | 124 | 124 (90) | 0.0149 |
| Barthel index on admission ≥80 | 171 | 48 (28) | 137 | 33 (24) | 0.43 |
| Medical history | | | | | |
| Hypertension | 177 | 32 (18) | 146 | 33 (23) | 0.31 |
| Diabetes | 177 | 15 (8.5) | 146 | 17 (12) | 0.34 |
| Dyslipidemia | 177 | 23 (13) | 146 | 18 (12) | 0.86 |
| Chronic kidney disease | 177 | 2 (1.1) | 146 | 7 (4.8) | 0.0429 |
| Laboratory findings on admission | | | | | |
| White blood cells/mm^3^ | 177 | 9700 (7285–12650) | 146 | 9850 (6638–14325) | 0.95 |
| Neutrocytes, % | 175 | 73 (65–80) | 143 | 83 (75–88) | <0.0001 |
| Lymphocytes, % | 177 | 17 (11–25) | 146 | 10 (6–16) | <0.0001 |
| Hemoglobin, g/dL | 175 | 14.1 (12.8–15.4) | 145 | 12.2 (11.2–13.7) | <0.0001 |
| Albumin, g/dL | 177 | 3.7 (3.5–3.9) | 146 | 3.0 (2.5–3.2) | <0.0001 |
| eGFR, mL/min/1.73 m^2^ | 177 | 60 (37–82) | 146 | 59 (36–77) | 0.32 |
| CRP, mg/dL | 177 | 2.9 (1.3–7.6) | 146 | 5.6 (2.1–11.4) | 0.0005 |
| BNP, pg/mL | 149 | 479 (230–854) | 120 | 768 (338–1370) | 0.0008 |
| NT-proBNP, pg/mL | 32 | 8338 (3051–20277) | 31 | 10816 (3832–26572) | 0.82 |
| PNI on admission | 177 | 46 (42–49) | 146 | 35 (31–38) | <0.0001 |
| LVEF, % | 175 | 33 (23–46) | 138 | 30 (20–43) | 0.13 |
| LVDd, mm | 145 | 46 (42–51) | 115 | 47 (42–50) | 0.54 |
| Treatment during hospitalization | | | | | |
| β-Blockers | 177 | 99 (56) | 146 | 91 (62) | 0.24 |
| ACE inhibitors or ARBs | 177 | 116 (66) | 146 | 90 (62) | 0.47 |
| Intravenous steroids | 177 | 74 (42) | 146 | 74 (51) | 0.11 |
| Intravenous immunoglobulin | 177 | 49 (28) | 146 | 50 (34) | 0.20 |
| Inotropes | 177 | 170 (96) | 146 | 141 (97) | 0.80 |
| Temporary MCS devices | | | | | |
| Intra-aortic balloon pumping | 177 | 137 (77) | 146 | 109 (75) | 0.57 |
| Venoarterial ECMO | 177 | 67 (38) | 146 | 65 (45) | 0.23 |
| Ventricular assist device | 177 | 6 (3.4) | 146 | 9 (6.2) | 0.24 |
| Events during hospitalization | | | | | |
| Ventricular tachycardia | 177 | 41 (23) | 146 | 35 (24) | 0.86 |
| Ventricular fibrillation | 177 | 22 (12) | 146 | 18 (12) | 0.98 |
| Advanced atrioventricular block | 177 | 48 (27) | 146 | 38 (26) | 0.82 |
| Histologically proven myocarditis | 177 | 93 (53) | 146 | 84 (58) | 0.37 |
| Length of hospital stay, day | 177 | 27 (17–47) | 146 | 33 (22–57) | 0.0155 |

Data were presented as numbers and percentages for categorical variables or median and first to third quartiles for continuous variables. BMI indicates body mass index; NYHA, New York Heart Association; eGFR, estimated glomerular filtration rate; CRP, C-reactive protein; BNP, brain natriuretic peptide; NT-proBNP, N-terminal pro-B-type natriuretic peptide; PNI, prognostic nutritional index; LVEF, left ventricular ejection fraction, LVDd, left ventricular end-diastolic diameter; ACE, angiotensin-converting enzyme; ARB, angiotensin II receptor blocker; MCS, mechanical circulatory support; and EMCO, extracorporeal membrane oxygenation.

**Supplementary Figure 1. Kaplan-Meire curve for the cardiovascular and non-cardiovascular death with the prognostic nutritional index at discharge.**

**
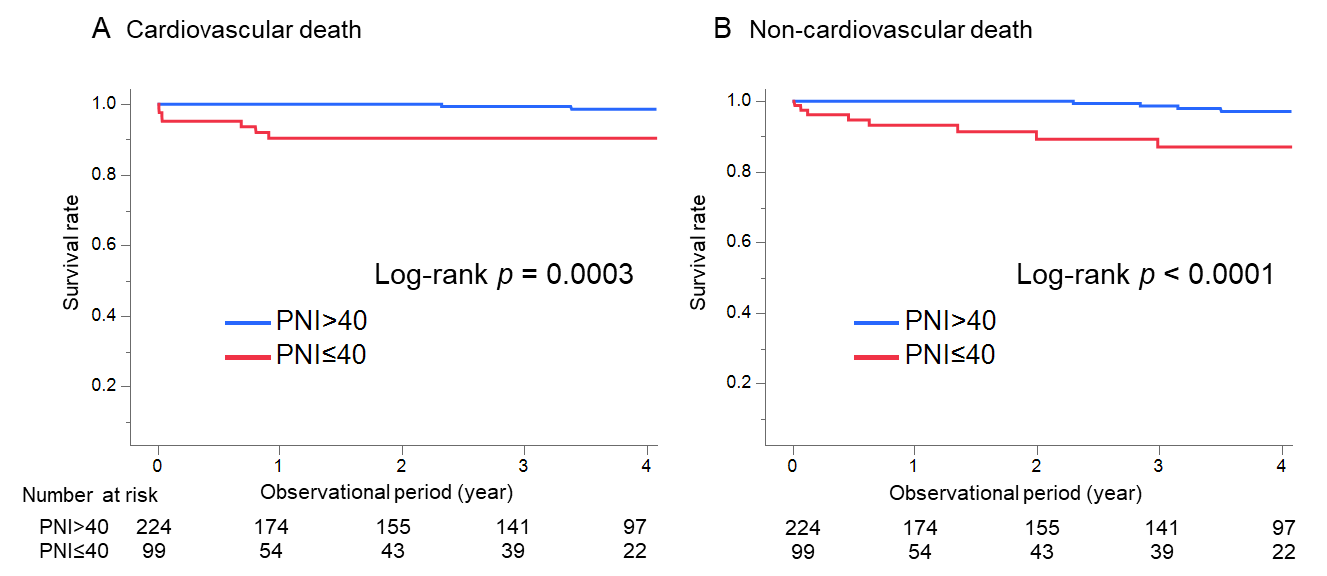
**

Survival rate with cardiovascular death (A) and non-cardiovascular death (B) between PNI ≤40 and PNI >40 at discharge.

PNI, prognostic nutritional index.

**Supplementary Figure 2. Comparison with the change in the component of the prognostic nutritional index at discharge.**


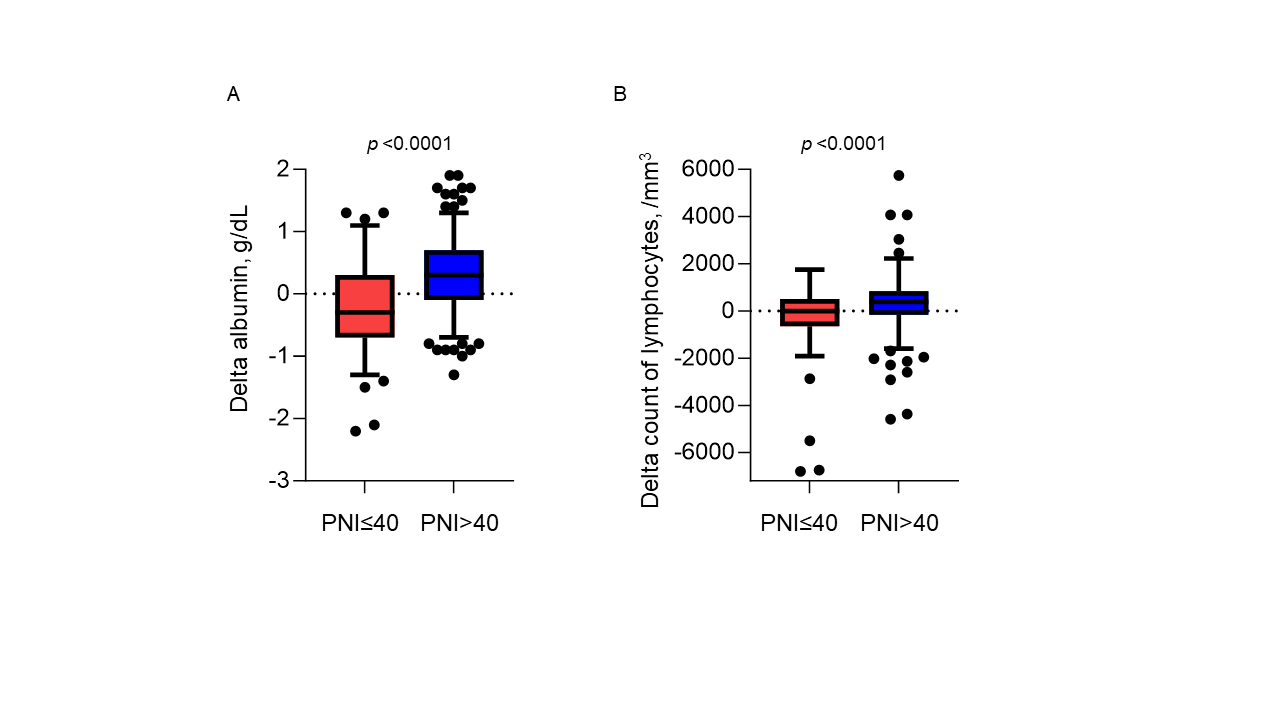
Patients with PNI ≤40 at discharge had more reduction of (A) serum albumin and (B) lymphocytes from admission than those with PNI >40. PNI, prognostic nutritional index.

**Supplementary Figure 3. Kaplan-Meire curve for the outcomes with the prognostic nutritional index on admission.**


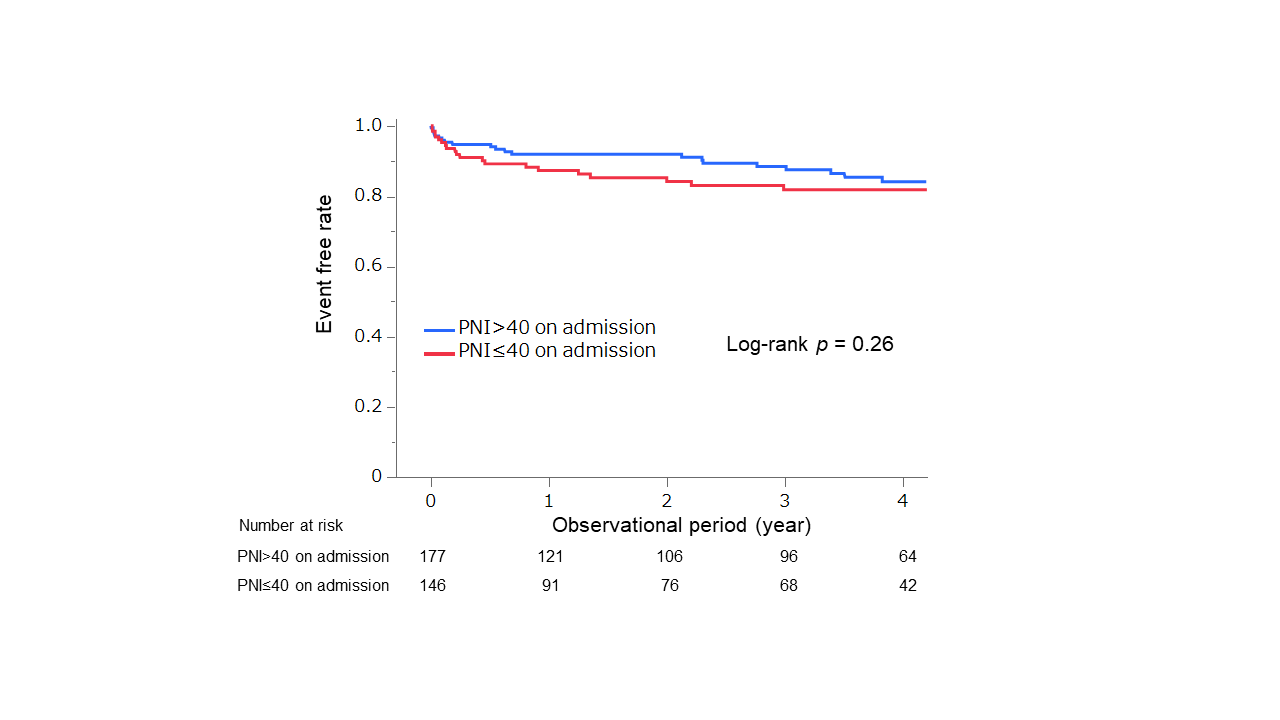


PNI, prognostic nutritional index.

**Supplementary Figure 4. Comparison of the prognostic nutritional index at discharge according to the histological characteristics.**


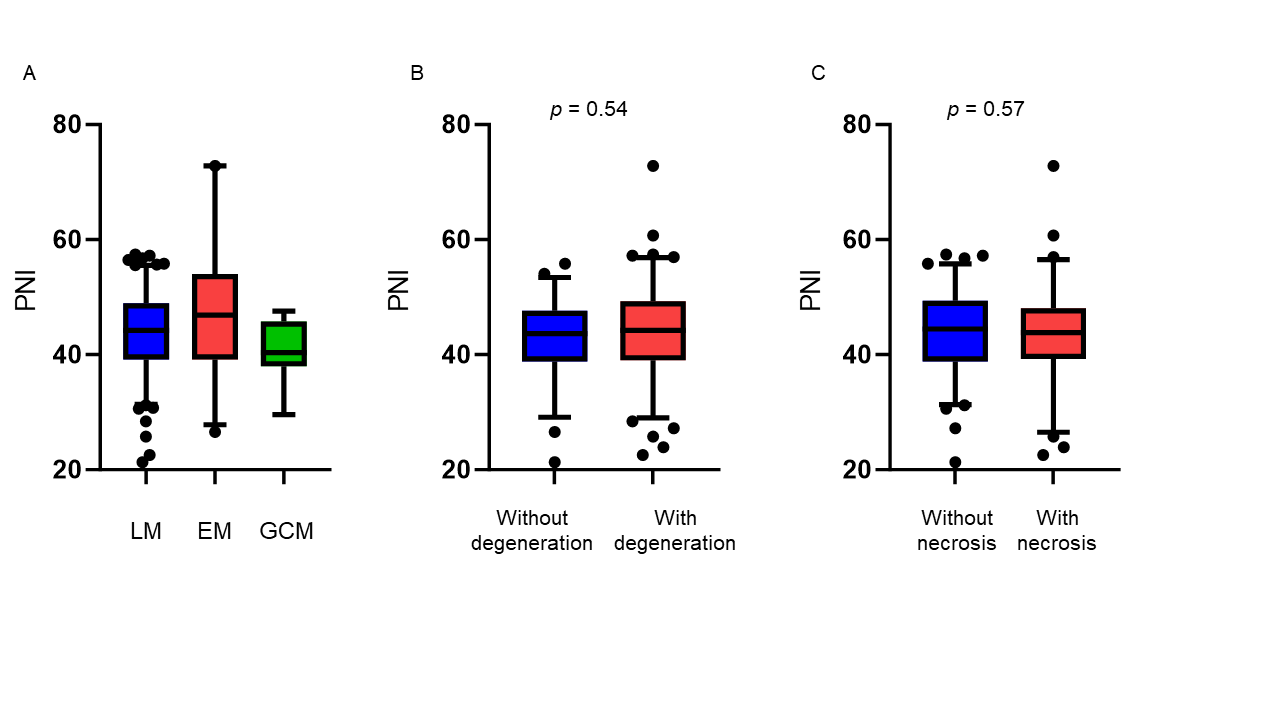


PNI at discharge was similar among (A) the histological subtype and severity, including (B) degeneration and necrosis. PNI, prognostic nutritional index; LM, lymphocytic myocarditis; EM, eosinophilic myocarditis; GCM, giant cell myocarditis.
